# Role of insulin-like growth factor binding protein-4 in prevention of colon cancer

**DOI:** 10.1186/1477-7819-5-128

**Published:** 2007-11-07

**Authors:** Rajaraman Durai, Shi Y Yang, Alexander M Seifalian, Geoffrey Goldspink, Marc C Winslet

**Affiliations:** 1 Academic Division of Surgical and Interventional Sciences, University College London, London, UK; 2 Royal Free Hampstead NHS Trust Hospital, London, UK

## Abstract

**Background:**

Insulin-like growth factors (IGFs) are important for the proliferation of cancer cells. One of their binding proteins, known as insulin-like growth factor binding protein -4 (IGFBP-4) is well known for its inhibitory action on IGFs *in vitro*. We assessed the effect of IGFBP-4 in prevention of development of colon cancer *in vivo*.

**Methods:**

Nude mice were subcutaneously inoculated with HT-29 colon cancer cells and they were also simultaneously injected either gene construct containing mammalian expression vector pcDNA3 with or without IGFBP-4 gene or phosphate buffered saline. The effect was assessed 4 weeks later by evaluating the tumours for mitosis, necrosis, apoptosis, and expressions of IGFBP-4, Bcl-2 and Bax proteins.

**Results:**

The results showed that the IGFBP-4 gene therapy did not prevent the tumour establishment but it increased the tumour apoptosis which was associated with an increase in Bcl-2 and Bax expressions. The IGFBP-4 protein was low in tumours which received IGFBP-4 gene construct which may be due to a feed back mechanism of IGFBP-4 upon its own cells.

**Conclusion:**

IGFBP-4 gene therapy in the form localised gene transfer did not prevent colon cancer initiation and establishment but it resulted in increased apoptosis and Bax protein expression and a decrease in tumour cellular mitosis

## Background

Colon cancer is the second leading cause of morbidity and mortality in the developed countries [1]. Several treatment options are available for established colon cancer depending upon the stage of the disease. No proven treatment option is available to date in preventing the establishment and development of colon cancer other than prophylactic total colectomy. Colon cancer development is influenced by several growth factors. Among these growth factors, insulin-like growth factor (IGF) system has been shown to play an important role in cancer development [2]. The IGF system consists of two growth factors (IGF-I and IGF-II), their binding proteins, receptors and proteases. One of the IGF binding proteins called IGFBP-4 is well known for its growth inhibitory effect on several cancer cells *in vitro *[3,4]. In our previous studies, we found that overexpression of IGFBP-4 on established subcutaneous cancer model can increase tumour apoptosis and decrease tumour cellular mitosis [5]. To explore the role of the IGFBP-4 in prevention of colon cancer establishment, we simultaneously administered gene construct containing IGFBP-4 cDNA at the same time when the subcutaneous cancer was induced in nude mice. The effect of IGFBP-4 on cancer initiation and development was then assessed by examining tumour volume, tumour histology, tumour cellular apoptosis and some proteins expression. In this paper we are presenting the results of our *in vivo *experiment.

## Methods

### Gene construct

Mammalian expression vector pcDNA 3 (Invitrogen, Carlsbad, CA, USA) containing IGFBP-4 gene, which was inserted between *Kpn *I and *EcoR *I restriction enzyme sites downstream of cytomegalovirus promoter, and light chain myosin enhancer was used. Plasmid DNA preparation and purification was performed using the Endo Free Plasmid Maxi Kit (Qiagen, Crawley, UK) and IGFBP-4 insert and its reading frame were confirmed by sequencing (MWG, Ebersberg, Germany) prior to animal experiment.

### Colon cancer cell culture

HT-29 human colon adenocarcinoma cells (European Collection of Cell Cultures, Porton Down, Dorset, UK) were cultured in 75 cm^3 ^flasks with McCoy's 5A Medium (GIBCO, Paisley, UK) containing glutamine (2 mM), 10% foetal bovine serum and 1% Penicillin (5000 unit/ml) and streptomycin (5000 μg/ml) at 37°C in an atmosphere of 5% CO_2_. 10 flasks of such cells were used. After 48 hours, the medium was extracted, the cells were washed twice with 10 mls of phosphate buffered saline (PBS) and then cultured in 20 mls of fresh medium under standard conditions until they reach to 90% confluent. Cells were then washed with 10 mls of PBS twice before they were trypsinised, neutralised with bovine serum and centrifuged at 900 G for 5 minutes. A final concentration of 12 mls of cells (5 × 10^6^/ml of >90% viability as determined by Tryphan Blue) was obtained and 0.6 mls (3 × 10^6^/ml cells in PBS) was injected subcutaneously into the flank of each nude mouse.

### Animal model

The experiment was conducted under a project licence, granted by the Home Office, UK, in accordance with the Animals (Scientific Procedures) Act 1986. 4–6 weeks old MF1 nu/nu male athymic mice were bought from Comparative Biology Unit, Royal Free and University College School, London, UK. The animals were randomly divided into three groups of 6 each. Group 1 received HT-29 cells alone in PBS (Control P), group 2 received a mixture of control plasmid and HT-29 cells (Control M) and group 3 received a mixture of HT-29 and gene construct containing IGFBP-4 cDNA (BP-4 group). Plasmids were used at 150 μg/animal in PBS and quantified prior to the animal experiment in UV spectrophotometer. The procedure was carried out under light enfluorane general anaesthesia and the cells/plasmid mixtures were injected into the flank subcutaneously with insulin syringes. The animals were kept in 4 per cage, each cage was separately marked and the animals in each cage were identified by ear punch. The experiment was terminated in 4 weeks after tumour induction. The tumour size was measured at two time points using a digital vernier caliper.

### Tumour volume

Four weeks after inoculation with cancer cells, the animals were sacrificed by schedule 1 method and tumours were harvested. Tumour volume was measured and calculated by a method described previously i.e. (the shortest diameter)^2 ^× (the longest diameter) × 0.5 [6]. The tumour tissues were divided into four portions and stored in appropriate medium for future assessment.

### Tumour histology

Paraffin sections were made from tumour samples which were fixed in 10% formalin. Haematoxylin and eosin (H&E) staining was carried out and the tumour sections were assessed for cell death and scored. Necrosis found in <1%, 1–20%, 21–40%, 41–60%, 61–80% and 81–100% of the region of interest were scored as 0, 1, 2, 3, 4 and 5 respectively [6,7]. Tumour proliferative activity was measured by counting the mitotic figures blindly in H&E stained tumour sections on 10 random high power fields (× 400) [8]. Mitotic figures in anaphase through early telophase were included in the counting.

### Assessment for apoptosis

Tumour apoptosis was investigated by both Terminal deoxynucleotidyl transferase biotin-dUTP nick end labelling (TUNEL) assay and electron microscopy.

#### TUNEL assay

TUNEL assay was carried out with Apotag-red kit (Serologicals Corporation, Temecula, CA, USA) to assess apoptosis. Tumour sections were fixed in 5% formalin for 10 minutes at room temperature and washed in PBS twice for 5 minutes per each wash. The manufacturer's protocol was then followed for the remaining step.

#### Electron microscopy

Transmission electron microscopy was performed to assess the ultra structure of the cancer cells to confirm and correlate the apoptosis with those of the TUNEL assay findings. Ultra thin sections (60–90 nm) were cut with a diamond knife and stained with uranyl acetate and lead citrate for examination. The tumour sections were viewed and ultra structures of cancer cell were photographed using a Philips CM120 transmission electron microscope.

### Assessment of IGFBP-4 by Western immunoblot

The frozen tumour tissues were powdered in liquid nitrogen and the cells were lysed in reporter lysis buffer (Promega, USA). Total proteins were quantified by modified Lowry protein assay (Pierce Biotechnology, Rockford, IL, USA). 50 μg of total protein from each tumour sample in 10 μl of PBS were mixed with an equal volume of laemmi (2× Sigma-Aldrich, Gillingham, UK) sample buffer. The samples were then denatured by heating in a water bath at 95°C for 5 minutes. Sodium dodecyl sulphate – polyacrylamide gel electrophoresis was used to separate the proteins, which was then electro blotted on to polyvinylidene fluoride (PVDF) membrane (Bio-Rad Lab, Hercules, CA, USA). The membrane was then blocked with 5% milk (Marvel semi skimmed milk powder, UK) for 30 minutes. The membrane was incubated with rabbit anti-IGFBP-4 polyclonal antibody (Santa-Cruz Biotechnology, Santa Cruz, CA, USA) at 1/200 dilution followed by incubation with Horse radish peroxidase (HRP) secondary antibody conjugate (Dako, Ely, UK) at 1/2000 dilution. The membrane was illuminated with Super Signal West Dura Extended Duration Substrate (Pierce, USA) and exposed to X-ray film for 10 seconds (Fuji, Japan). The X-ray was scanned and the density of the protein bands were analysed with Bio-rad densitometry software (Molecular Analyst, Windows software for Bio-rad's image analysis system version 1.5, USA).

### Assessment of Bax and Bcl-2 expressions by Western blot

The procedure for Western blot is the same as for the IGFBP-4. Rabbit anti-mouse Bcl-2 polyclonal antibody (Santa-Cruz Biotechnology, Santa Cruz, CA, USA) or rabbit anti-mouse Bax polyclonal antibody (Santa-Cruz Biotechnology, USA) both at 1/200 dilution were used, followed by incubation with secondary antibody conjugate with horse radish peroxidase (HRP) (Dako, Ely, UK) at 1/2000 dilution.

### Statistics

One way ANOVA with Dunnett post hoc test (Graph pad, Prism version 4 2004 edition, USA) was used for statistical calculations and P <0.05 was considered as significant.

## Results

### Tumour establishment, volume and weight

**Figure 1:**
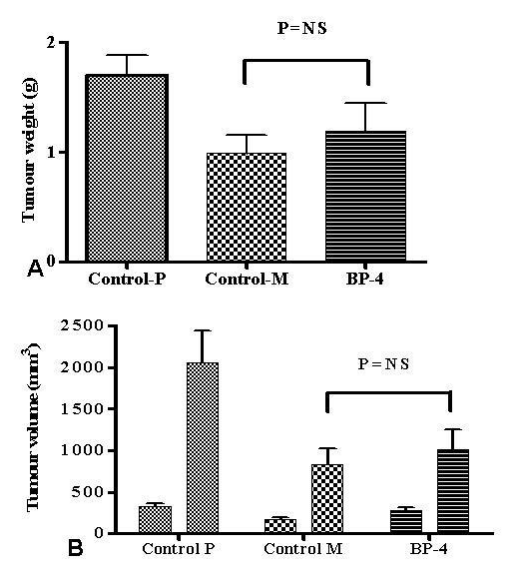
Showing final weight (a) and tumour volumes on week 1 and 4 after tumour induction (b) of tumours of BP-4 treated, control M and control P groups. There was no statistical difference between control M and BP-4 treated tumours. Values are expressed as mean ± SEM.

All animals developed subcutaneous tumours. There was no local or distant spread of cancer. Both tumour weight and volume showed a reduction in animals which received plasmid treatment although there was no statistical difference in the final tumour weight (grams) (1.70 ± 0.17 vs 0.98 ± 0.16 vs 1.19 ± 0.25; Control P vs Control M vs BP-4; P = 0.06) (figure 1a) or tumour volume (mm^3^) (2060.25 ± 379.62 vs 829.99 ± 194.81 vs 1007.71 ± 246.67; Control P vs Control M vs BP-4; P = 0.69) (figure 1b) between Control and BP-4 group.

### Tumour histology

**Figure 2:**
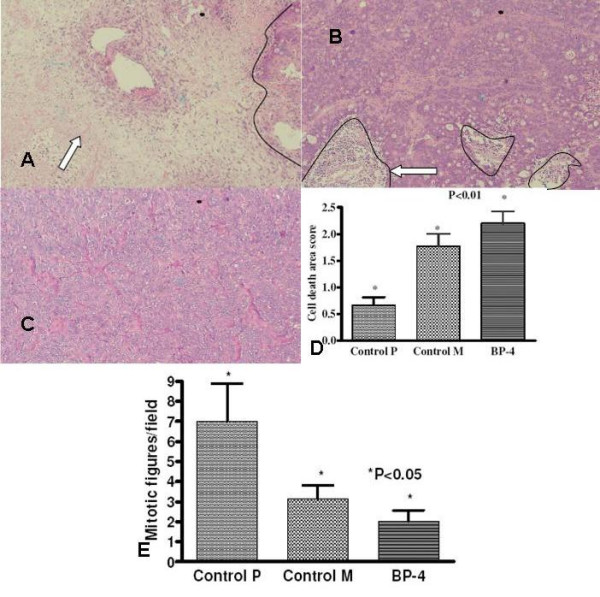
Photomicrographs (×200) showing H&E staining of tumours of (a) BP-4 (b) control M (c) control P groups. Increased areas of necrosis (arrow) and decreased cellular density are present in BP-4 treated tumours when compared to control groups. (d) Showing cell death score of tumours. The animals received a single administration of either saline or gene construct with and without BP-4 along with colorectal cancer cells. Results are shown as mean ± SEM of 6 animals in each group. (e) Showing average numbers of mitotic figures per section per field of tumours. Values are expressed as mean ± SEM of 6 animals in each group. (*key: P – animals without plasmid therapy, M – animals which received control plasmid, BP-4 – animals which received IGFBP-4 gene.)*

There was an attempted glandular formation with areas of cell death. The mean area of cell death was significantly higher in BP-4 group tumours than the other two control group tumours (2.20 ± 0.22 vs 1.76 ± 0.23 vs 0.66 ± 0.14, BP-4 vs Control M vs Control P, P = 0.0007). Figures 2a, b and 2c show the representative photomicrographs of tumours after H&E staining in each group and figure 2d shows the cell death score. The average numbers of mitotic figures per field were lower in BP-4 group tumours than the control group tumours (2.03 ± 0.53 vs 3.13 ± 0.99 vs 7.00 ± 1.72, BP-4 vs Control M vs Control P, P = 0.03) (figure 2e).

### Tumour apoptosis

**Figure 3:**
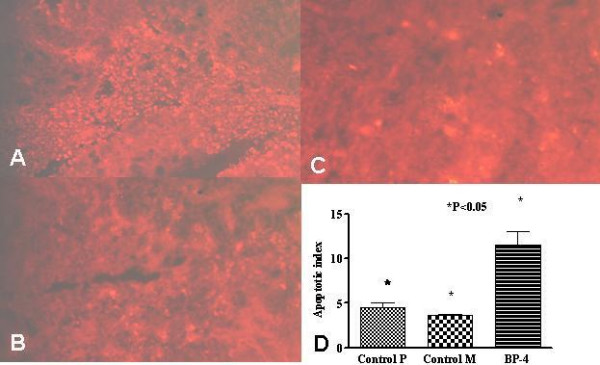
Representative photomicrographs showing TUNEL assay of tumours (× 200) from (a) BP-4 group (b) control M and (c) control P groups. Apoptotic cells were detected by red immunofluorescence. BP-4 treated tumours show increased numbers of apoptotic cells (arrow) compared with the other two groups (d) Comparison of apoptotic indices among the tumours of BP-4, control M and control P groups. BP-4 group tumours had higher apoptotic index than control groups. Values are shown as mean ± SEM of 6 animals in each group.

**Figure 4:**
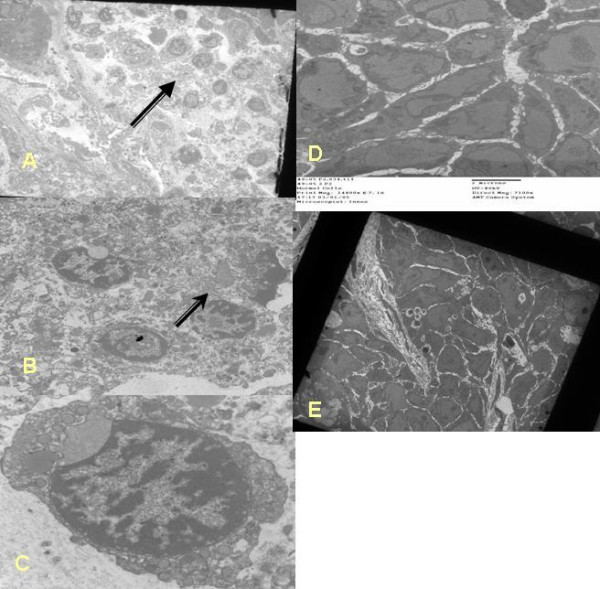
**(a) **Electron microscopic image of tumours of BP-4 group at a magnification of × 710. Obviously there are numerous apoptotic cells with condensed nucleus (arrow) **(b)**Transmission electron microscopic imaging showing apoptotic cells which are seen as apoptotic bodies (arrow) with a shrunken nucleus and a condensed cytoplasm in BP-4 treated tumours (×11500)**(c)**Transmission electron microscopic imaging (×11500) showing apoptotic cells with dense masses of chromatin against nuclear membrane in BP-4 group tumours **(d) **Electron microscopy of control P group tumours showing necrotic cells (**e) **Transmission electron microscopic image of control M treated tumours (× 710) showing no apoptotic cells.

TUNEL assay demonstrated higher numbers of apoptotic cells in tumours of the BP-4 group than the control groups (figure 3). Apoptotic index is plotted in figure 3d. It showed that IGFBP-4 gene therapy treatment resulted in higher concentration of apoptotic cells in tumours when compared to control group tumours (apoptotic cells/100 total cells) (11.47 ± 1.51 vs 3.59 ± 0.17 vs 4.47 ± 0.55, BP-4 vs Control M vs Control P, P = 0.0002). Transmission electron microscopy (figure 4a–e) also demonstrated higher numbers of cells undergoing apoptosis in tumours of BP-4 group while less numbers of such cells were noted in tumours of control groups.

### IGFBP-4 protein expression

**Figure 5:**
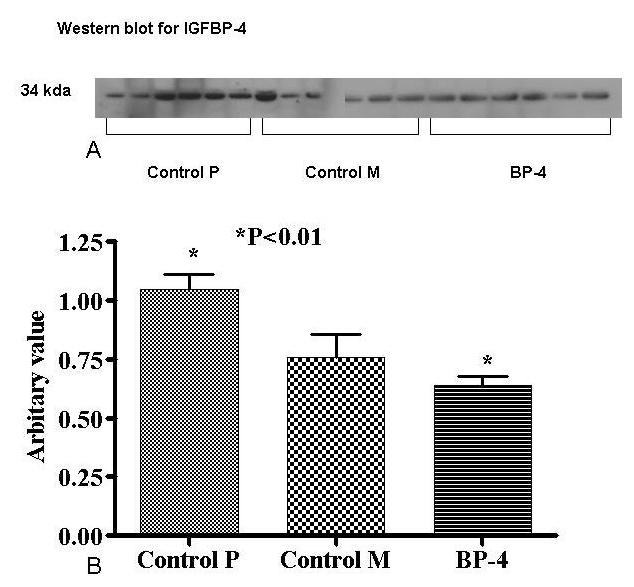
Western blot for IGFBP-4 expression (a) and densitometry analysis (b) of subcutaneous tumours showing higher expression of IGFBP-4 by Control M than the other two groups (P < 0.05). Values are expressed as mean ± SEM of 6 animals in each group. *(Key: control P – tumours without any plasmid treatment, control M – tumours receiving control plasmid therapy, BP-4 – tumours receiving plasmid with BP-4 construct)*

Western blot and densitometry analysis (figure 5) showed a decreased expression of IGFBP-4 by tumours of both BP-4 and control M groups and an increased expression by Control P group (0.63 ± 0.03 vs 0.75 ± 0.09 vs1.04 ± 0.06, BP-4 vs control M vs Control P, P = 0.002).

### Expression of Bax and Bcl-2 Proteins

**Figure 6:**
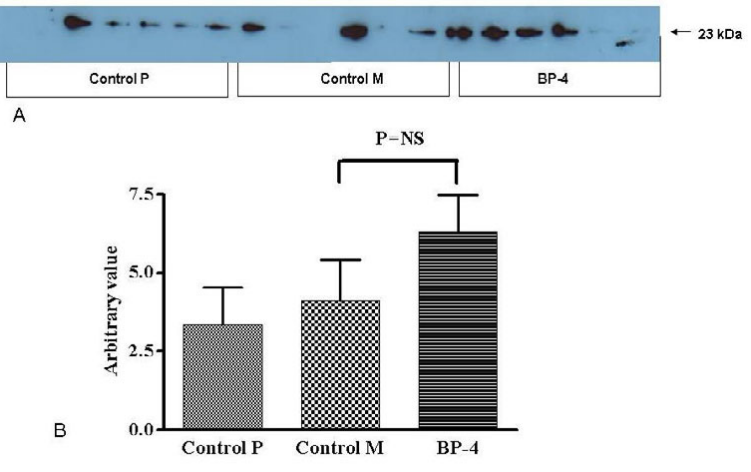
Western blot (a) and densitometry analysis (b) for Bax protein by subcutaneous tumours. BP-4 group tumours showed higher expression of Bax protein compared to control groups P and M. Values are shown as mean ± SEM. (*NS = not significant)*

**Figure 7:**
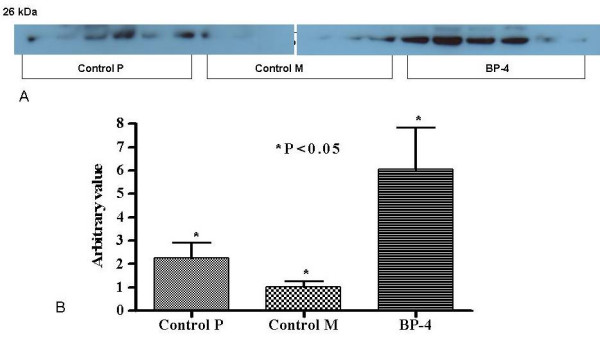
Western blot (a) and densitometry analysis (b) of Bcl-2 protein in tumours receiving gene constructs with (BP-4) or without IGFBP-4 cDNA (control C) or no gene constructs (control P). The result showed a higher expression of Bcl-2 by BP-4 group compared to Control P and M. Values are shown as mean ± SEM of 6 animals in each group.

The Bax protein was over-expressed in tumours after IGFBP-4 gene therapy when compared with control groups (3.37 ± 1.15 vs 4.11 ± 1.30 vs 6.31 ± 1.16; Control P vs Control M vs BP-4; P = 0.23) (figure 6). The mean Bcl-2 expression was higher (2.27 ± 0.65 vs 1.01 ± 0.25 vs 6.06 ± 1.77, Control P vs Control M vs BP-4, P < 0.01) in BP-4 treated tumours when compared with the control group (figure 7).

## Discussion

In this study we assessed the preventive role of IGFBP-4 in colon cancer in the form of gene therapy *in vivo *and found that IGFBP-4 did not prevent the establishment of cancer in HT-29 colon cancer model but it increased the cell death, apoptosis and decreased tumour proliferation. This increase in apoptosis was associated with an increase in Bax protein expression.

Apoptosis is a mechanism of single cells death in which mitochondria play an important role. During apoptosis, cells shrink in their size and there is condensation of chromatin resulting in the formation of apoptotic bodies [9]. It is characterised by extensive DNA fragmentation [10] with no accompanying inflammation. In contrast to this, necrosis results in cellular swelling and there is inflammatory response.

In our experiment, histopathological examination of tumour tissue showed increased areas of cell death and fewer mitotic figures in BP-4 group tumours when compared to control group tumours. TUNEL assay and electron microscopy both confirmed these dead cells as apoptotic cells. Apoptotic index was significantly higher in tumours of BP-4 group when compared with controls. Apoptosis is influenced by various intracellular proteins and enzymes. IGFs, cause cellular proliferation and by their anti apoptotic action [11], prolong cell survival. IGFs act on IGF-IR which in turn alters various intra cellular proteins and enzymes. Apoptosis is influenced by the actions of various intracellular pro-apoptotic and anti- apoptotic proteins. The pro-apoptotic proteins belong to Bax subfamily and anti-apoptotic proteins belong to Bcl-2 subfamily. IGF-I acts at different control points of apoptosis, including the Bcl-2 family proteins, inhibitors of caspases and signalling of death-inducing receptors [12]. Bax protein plays an important role in cellular apoptosis [13]. It can form a transmembrane pore across the outer mitochondrial membrane, leading to loss of membrane potential and efflux of cytochrome c and apoptosis inducing factor. IGF-I not only down regulate the Bax expression [14] but also prevent its translocation to mitochondria [15], inhibits the activation of caspase 3 [16,17] and release of cytochrome c from the mitochondria [16]. Bcl-2 inhibits the release of cytochrome c from the mitochondria and thereby it may influence apoptosis.

In our previous study [5,18], we assessed the effect of local IGFBP-4 gene therapy on a previously established subcutaneous cancer model and found that the IGFBP-4 and IGF-1R were overexpressed by the tumours. There was an associated increase in Bax and a decrease in Bcl-2 proteins after the gene therapy. TUNEL assay demonstrated an increase in apoptotic index by the tumours after IGFBP-4 gene therapy. However, the tumours did not regress after gene therapy.

In our current study, tumour volume and weight were similar in both BP-4 and Control M groups. However the microscopic parameters were different. This may mean that macroscopic parameters are not reliable indicators of response to IGFBP-4 gene therapy. This tumour model is not a survival model. Therefore the long-term outcome of the gene therapy and effect on animal survival is not known. As per Home Office guidelines the animals were sacrificed by Schedule 1 method even before they become moribund and the tumour attains 2 cm. The expression of IGFBP-4 was higher in tumours of control P group than other two groups. It indicates that the IGFBP-4 might have been used up or prior establishment of tumour could be a pre requisite for IGFBP-4 expression. Another possible explanation is tumour cells when deprived of IGF-I for a long time may be unable to produce IGFBP-4. Both Bcl-2 and Bax expressions were increased in BP-4 group tumours. IGF-1R expression could not be detected. The reason for Bcl-2 up regulation after IGFBP-4 gene therapy is unclear. Perhaps Bcl-2 may have some inverse relation with IGFBP-4.

This study showed that IGFBP-4 gene therapy did not prevent the establishment of colon cancer from HT-29 cells in nude mice but it resulted in an increase in apoptosis indicating IGFBP-4 may influence tumour growth or development but not cancer initiation. There was an associated increase in Bax protein suggesting that it may be the mechanism of apoptosis.

## Conclusion

From our experiment, we conclude that IGFBP-4 gene therapy in the form localised gene transfer did not prevent colon cancer initiation and establishment but it increased in apoptosis and Bax protein expression and a decrease in tumour cellular mitosis. Further experiments are needed to find out whether the IGFBP-4 gene therapy can be combined with chemotherapeutic agents in preventing the establishment of colon cancer in situations such as familial adenomatous polyposis.

## Competing interests

The author(s) declare that they have no competing interests.

## Authors' contributions

**RD **Wrote this manuscript and is the first author

**SY **Guided RD in writing up the manuscript

**AS **Overall supervision of the experiment in terms of design and methodology

**MW **&**GG **– Senior authors who corrected the manuscript and helped in revision of the contents and data analysis
